# Oyster Cultivation in France

**Published:** 1874-11

**Authors:** 


### OYSTER CULTIVATION IN FRANCE.

Formerly France possessed a great abundance of native oysters. But this industry was without regulation, and the French natives, like our Northern natives, came near being exterminated. A few years ago Prof. Coste, of the French Academy, called attention to the fact that the French oyster was becoming extinct. He took up the study of this mollusk in earnest, and learned many important facts concerning its nature. He even went to the Neapolitan oyster park, and observed how the fishermen there saved the young ones. He then appealed to the government, which put means in his way for experimenting, and, in a short time, he had a successful oyster plantation under way. It is in France as elsewhere; "seeing is believing," and "there is nothing that succeeds like success." Under the wise direction of this learned naturalist, the new industry, oyster-planting, became a furor in France. "In two years 1,200 capitalists, associated with a similar number of fishermen, occupied a surface of 988 acres." By which is meant the area of short-line exposed at low tide. And what labor! so thorough and scientific. The isle of Re, with its unsuitable, muddy shores, had all that sea-bottom altered. In two years twelve miles of sea-coast thus changed was planted, with 1,200 parks in operation, and thousands more projected. Now, oyster-culture is conducted in France on better principles than anywhere else. And all of this great additional wealth to the nation comes out of the applied science of a man "that studied shells and worms," as is often said in derision. In France scrupulous provision is made for husbanding the fry. In America no effort is made in this direction, and the time is not far off when the nation will wake up to a serious calamity in this respect.—*Popular Science Monthly for November.*

